# Two new species and distribution records for the genus *Bohayella* Belokobylskij, 1987 from Costa Rica (Hymenoptera, Braconidae, Cardiochilinae)

**DOI:** 10.3897/zookeys.996.59075

**Published:** 2020-11-24

**Authors:** Ilgoo Kang, Scott R. Shaw, Nathan P. Lord

**Affiliations:** 1 Department of Entomology, Louisiana State University Agricultural Center, 404 Life Sciences Building, Baton Rouge, LA, 70803, USA Louisiana State University Agricultural Center Baton Rouge United States of America; 2 UW Insect Museum, Department of Ecosystem Science and Management (3354), University of Wyoming, 1000 E. University Avenue, Laramie, WY 82071, USA University of Wyoming Laramie United States of America

**Keywords:** Morphology, New World, parasitoid wasp, taxonomy

## Abstract

Two new species of *Bohayella* Belokobylskij, 1987 from Costa Rica are described: *Bohayella geraldinae* Kang, **sp. nov**. and *Bohayella hansoni* Kang, **sp. nov.** These are new distribution records for the genus in the Neotropical region. In addition, a key to species of the genus *Bohayella* of Costa Rica is presented. The current work elevates the number of species included in *Bohayella* from nine to eleven.

## Introduction

Costa Rica is one of the biodiversity hotspots, and a total estimated hymenopteran fauna in the country is ~ 20,000 species, including ~ 2,000 estimated species of braconid wasps (Gaston et al. 1996). Cardiochilinae is a subfamily of Braconidae, containing 17 genera and 220+ species (Yu et al. 2012; Kang et al. 2020). *Bohayella* Belokobylskij, 1987 (Belokobylskij 1987) is an unusual genus of the subfamily, with nine previously described species that are only known from the Old World, including Afrotropical, Australasian, Oriental, and southern central Palearctic regions (Dangerfield et al. 1999; Mercado and Wharton 2003; Yu et al. 2012). Among the nine Old World species of *Bohayella*, two species, *B. adina* (Wilkinson, 1930) and *B. exiguurus* (Huddleston & Walker, 1988), have rearing records (Huddleston and Walker 1988). *B. adina* was reared from larvae of *Phazaca theclata* (Guenée, 1857) (Lepidoptera: Uraniidae) in India, (Beeson and Chatterjee 1935; Dangerfield 1995; Dangerfield et al. 1999), and *B. exiguurus* was reared from larvae of the citrus looper *Cleora tulbaghata* (Felder & Rogenhofer, 1875) (Lepidoptera: Geometridae) South Africa (Dangerfield et al. 1999).

*Cardiochiles nigricans* Mao, 1949 (Mao 1949) was transferred into *Bohayella* by Dangerfield et al. (1999) and recorded as the first species of *Bohayella* in the New World. Mercado and Wharton (2003) transferred the species into *Toxoneuron* Say, 1836 because the first metasomal tergite (T1) of the species is different from T1 of other members of *Bohayella*. Subsequently, members of *Bohayella* have been restricted to the Old World and new species of the genus have not been reported from the New World.

The first author (IK) had the opportunity to examine Costa Rican cardiochiline specimens housed in University of Wyoming Insect Museum (UWIM). Using the key to world genera of the subfamily Cardiochilinae and other diagnostic characters of *Bohayella* (Dangerfield et al. 1999), nine *Bohayella* specimens were identified. The characters of New World *Bohayella* are discussed in detail in diagnosis and discussion sections of this paper. Other Costa Rican cardiochiline specimens borrowed from several institutions were examined, but no more specimens of *Bohayella* were discovered. As a result, the nine specimens of *Bohayella* were confirmed as two species based on morphological data. Herein, we describe two new species and present a key to species of the genus *Bohayella* of Costa Rica. Distribution maps for both species are included.

## Materials and methods

Specimens for this project were provided by **UWIM** (University of Wyoming Insect Museum; 1000 East University Avenue, University of Wyoming, Laramie, Wyoming 82071-3354, USA). We conducted morphological analyses using a Leica MZ75 stereomicroscope. The morphological terms and terms of wings mostly follow Dangerfield (1995) and Dangerfield et al. (1999). Morphological terminology can be checked at the Hymenoptera Ontology website (http://portal.hymao.org/projects/32/public/ontology/) as well. Terms for sculpturing are based on Harris (1979). Color habitus images were taken using a Visionary Digital BK Plus imaging system (Dun, Inc.), equipped with a Canon EOS 5DS DSLR camera. Images were stacked *via* Zerene Stacker v.1.04 (Zerene Systems LLC.). All images were edited using Adobe Photoshop CS 6 (Adobe Systems, Inc). Body parts of each species were measured via Adobe Photoshop CS 6 (Adobe Systems, Inc). Each number in parentheses in species descriptions indicate 0.01 times the actual length, width, or height of each body part. For example, 42 and 124 in parentheses (42:124) indicate 0.42 mm and 1.24 mm, respectively. Distribution maps of two *Bohayella* species were produced using QGIS 3.10.0 (QGIS Development Team 2019). Google satellite maps were downloaded using the QuickMapServices plugin. The following abbreviations are used throughout the current paper: POL: distance between posterior ocelli, T1, T2 (second metasomal tergite), T3 (third metasomal tergite), T5 (fifth metasomal tergite), T6 (sixth metasomal tergite), T7 (seventh metasomal tergite), and T8 (eight metasomal tergite). Holotypes and paratypes are deposited in the UWIM.

## Results

### Bohayella

Taxon classificationAnimaliaHymenopteraBraconidae

Belokobylskij, 1987

092F2980-291B-58DF-A0E1-17D7C0E61868

#### Type species.

*Bohayella tobiasi* Belokobylskij, 1987.

#### Diagnosis

**(based on Dangerfield et al. (1999) with modifications and additions).** Diagnostic characters of *Bohayella* based on Old World members were described in Belokobylskij (1987) (in Russian) and Dangerfield et al. (1999) (in English). The following are re-described or additional characters based on morphological characters of both Old World and New World members.

Members of the genus can be identified by setose compound eyes (length and density variable); ventro-posteriorly moderately extended gena (Fig. 3F).broad clypeus without clypeal tubercles (Figs 3B, 4C); absence of occipital carina; uni- or bi-dentate mandible; 5- or 6-segmented maxillary palpus; 4-segmented labial palpus; short mouthparts (galea and glossa); deep and broad notauli and scutellar sulcus (Figs 3C, F, 4B, D); scutellum with apical cup-like pit (Figs 3C, F, 4B, D); fully developed propodeal areola (Figs 3D, F, 4B, E); moderately to strongly sculptured pronotum and mesopleuron; presence of epicnemial carina (Figs 3A, 4A); well-defined and crenulate precoxal sulcus (Figs 3A, 4A); absence of apical cup-like projection of hind tibia (Figs 3A, 4A); cylindrical or antero-posteriorly slightly expanded hind basitarsus (but never expanded like hind basitarsi found in members of *Hartemita* Cameron, 1910) (Figs 3A, 4A); pectinate tarsal claw with sharp or obtuse apical tooth; entirely or apically infuscate forewing; absence of 1r vein of forewing; absence of 3r vein of forewing; basally angled or smoothly curved Rs vein; absence of 2-1A vein of hind wing; narrow and elongate T1 (median length of T1 4.0–6.3× longer than its apical width) (Figs 3D, F, 4B, E); short T2; a medio-basal ball-like projection of T2 (Figs 3D, F, 4B, E); short and truncate hypopygium (Figs 3A, 4A); short ovipositor (if protruded, strongly downcurved); short ovipositor sheath (< ~0.2× longer than hind tibia) (Figs 3A, 4A).

### Key to species of the genus *Bohayella* of Costa Rica

**Table:** 

1	Median crenula of notauli as long as median crenula of scutellar sulcus (A); scutellar sulcus with one median crenula (A); T3–T8 mostly pale (AA)	***B. geraldinae* sp. nov.**
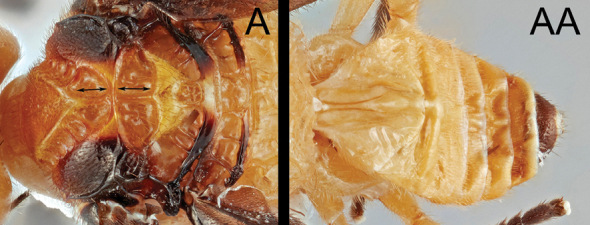		
–	Median crenula of notauli shorter than median crenula of scutellar sulcus (B); scutellar sulcus with three crenulae (B); T3–T8 mostly melanistic (BB)	***B. hansoni* sp. nov.**
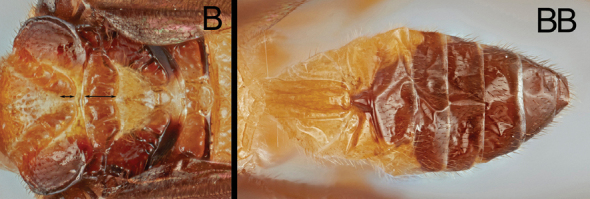		

### Bohayella geraldinae

Taxon classificationAnimaliaHymenopteraBraconidae

Kang sp. nov.

E80E167A-E9EA-56D1-B74F-036C4920D938

http://zoobank.org/FC39B76A-3AC3-41D7-9420-7148410F6D2C

Figure 3

#### Material examined.

***Holotype*** Costa Rica • ♀; female, Heredia, 3 km S. Puerto Viejo OTS, La Selva; 100 m; x.1992; P. Hanson; huertos Malaise trap set by G. Wright. ***Paratypes*** Costa Rica • 1 ♀; same data as for holotype; xi.1992 • 1 ♂; male; same collecting data as for preceding; 10°26'N, 84°01'W; 4. iv. 1987; H. A. Hespenheide.

#### Diagnosis.

*Bohayella geraldinae* sp. nov. can be recognized by the following combination of characters: apical maxillary palpomere as long as fifth maxillary palpomere; median crenula of notauli as long as median crenula of scutellar sulcus; scutellar sulcus with one median crenula; hind basitarsus antero-posteriorly slightly expanded; dorsal metasoma mostly pale.

#### Description.

**Female.** Body 4.6–4.8 mm. Forewing length: ~ 4.2 mm Antenna length: ~ 4.8 mm. ***Head*.** Antenna 34-segmented. Interantennal space with well-developed median carina. POL ~ 1.38× longer than diameter of anterior ocellus (11:8) (Fig. 3F). Eye sparsely setose with short setae; median width of eye 0.75× longer than median width of gena in lateral view (36:48). Width of clypeus ~ 2.07× longer than height (60:29) (Fig. 3B). Malar space ~ 2.62× longer than basal width of mandible (34:13) (Fig. 3B). Mandible bidentate. Maxillary palpus 6-segmented; apical maxillary palpomere as long as fifth maxillary palpomere. ***Mesosoma*.** Mesoscutum with sharp margin (Fig. 3C, F). Notauli broadly converging at base, with 11 crenulae; median crenula of notauli as long as median crenula of scutellar sulcus (Fig. 3C, F). Scutellar sulcus with one median crenula (Fig. 3C, F). Postscutellar depression present (Fig. 3C, F). Propodeum rugulose with well-defined median areola; median transverse carina on propodeum reaching lateral margin (Fig. 3D, F). Pronotum dorso-posteriorly crenulate and antero-ventrally smooth. Mesopleuron dorsally and posteriorly with crenulate margin (Fig. 3A). Mesosternal sulcus broad and crenulate. Metapleuron rugulose. ***Legs*.** Basal spur on fore tibia ~ 0.86× longer than basitarsus (30:35). Width of hind femur ~ 0.34× longer than its length (42:124). Basal spur on hind tibia ~ 0.76× longer than basitarsus (58:76). Hind tarsal claw pectinate, with four sharp teeth (Fig. 3E). ***Wings*.** Forewing second submarginal cell trapezoidal, ~ 0.35× longer than maximum width (30:85); 3r absent (Note: one specimen has basally present 3r vein in particular angle); Rs sharply angled at basal third; stigma ~ 2.67× longer than medial width (80:30). 1CUa short, 0.23× longer than 1CUb (12:52). Hind wing 2-1A absent. ***Metasoma*.**T1 with a pair of lateral sutures posteriorly reduced, median length of T1 ~ 5.07× longer than apical width (71:14) (Fig. 3D, F). T2 with a ball-like projection, medially 0.21× longer than T1 (15:71) (Fig. 3D, F). T3 ~ 2.13× longer than T2 medially (32:15) (Fig. 3D, F). Protruded ovipositor sheath ~ 0.13× longer than hind tibia and apically setose (20:154) (Fig. 3A).

**Male.** Body ~ 5.0 mm. Same as female except for the following characters: antenna 32-segmented, melanistic color does not reach the dorsal margin of foramen magnum.

**Color.** Body mostly pale; the following areas are melanistic: antenna, vertex, frons, dorsal occiput, maxillary palpus, labial palpus, lateral mesonotal lobe (pale basally), lateral scutellum, margin of metanotum, apical fore femur, fore tibia, apical fore tarsus, apical mid femur, mid tibia, apical mid tarsus, apical hind femur, basal and apical hind tibia, apical hind tarsus, posterior T5 and T6 (weakly), entire T7 and T8, ovipositor sheath. Wings entirely infuscate, stigma entirely melanistic.

#### Host.

Unknown.

#### Distribution.

**Figure 1.**
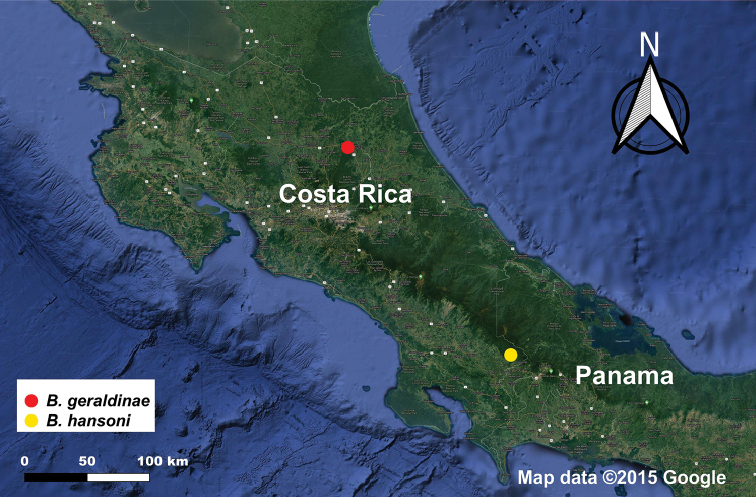
Distribution map of the species of *Bohayella* in Costa Rica. Map data 2020 Google.

**Figure 2.**
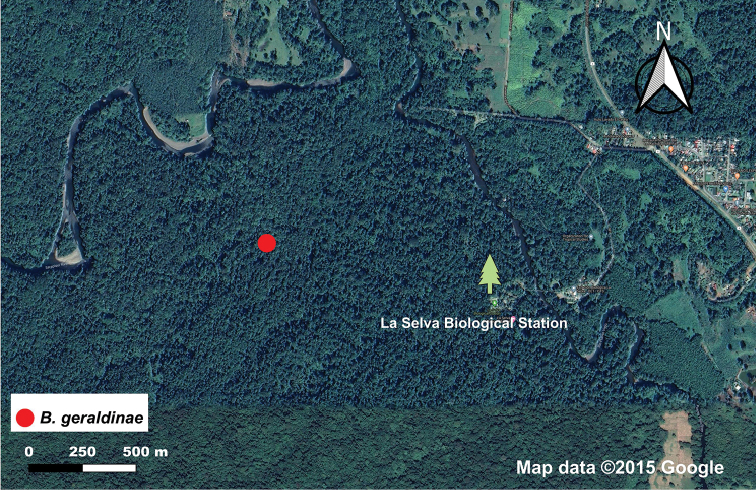
Distribution map of *B. geraldinae* sp. nov. in La Selva Biological Station in Costa Rica. Map data 2020 Google.

*Bohayella geraldinae* sp. nov. is known only from the La Selva Biological Station owned and managed by Organization for Tropical Studies (OTS) in Heredia, Costa Rica at an elevation of 100 m (Figs 1, 2). The station is located in the Caribbean lowlands, at a confluence of the Sarapiquí river and Puerto Viejo (McDade and Hartshorn 1994). According to Holdridge’s life zone system (Holdridge 1967), the station is in the tropical wet forest region (Hartshorn and Peralta 1987), and the average annual precipitation in the area is ~ 4,000 mm (Sanford et al. 1994).

**Figure 3.**
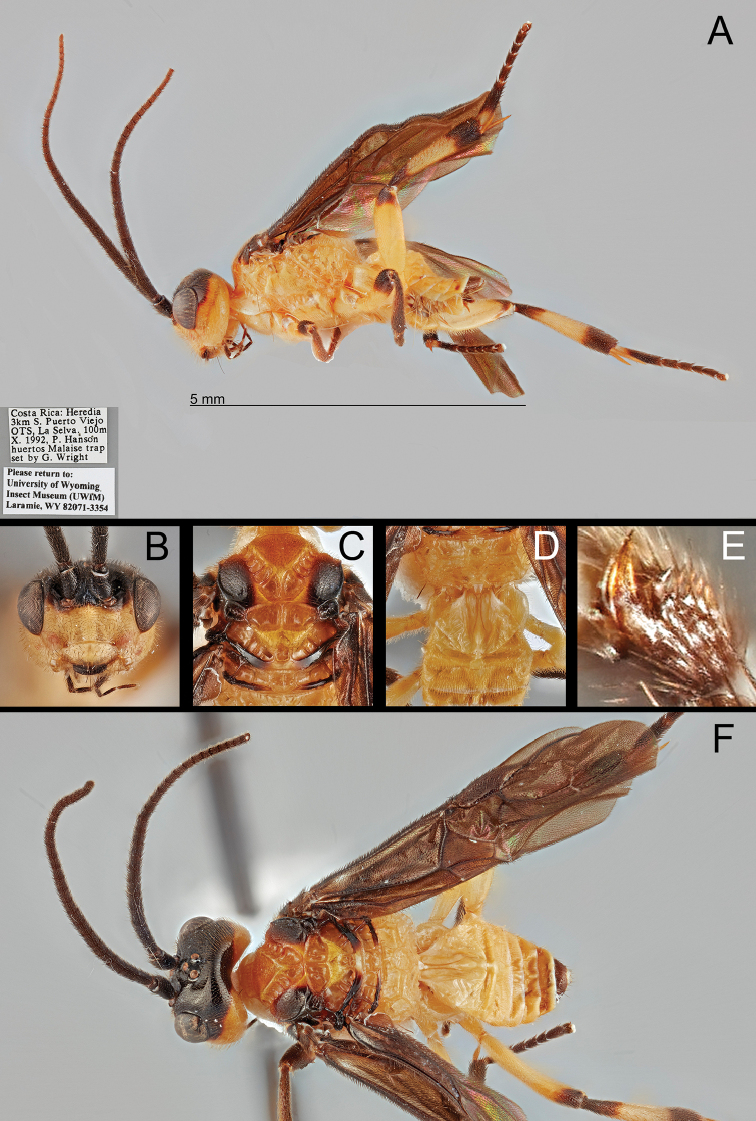
*B. geraldinae* sp. nov., holotype **A** lateral habitus **B** anterior head **C** dorsal mesonotum **D** dorsal propodeum and T1–T3**E** hind tarsal claw **F** dorsal habitus.

#### Etymology.

This species is named in honor of Dr Geraldine Wright, a former student of the second author (SRS), Rhodes Scholar, professor in the Department of Zoology in the University of Oxford (United Kingdom), and the person who set the trap that collected the specimens.

### Bohayella hansoni

Taxon classificationAnimaliaHymenopteraBraconidae

Kang sp. nov.

B8B4FC84-1DF0-546A-865E-7321579F6150

http://zoobank.org/7749425B-2B7F-4E69-A115-B65ED9CAD0CF

Figure 4

#### Material examined.

***Holotype*** Costa Rica • ♀; female, Puntarenas, San Vito, Estac. Biol., Las Alturas; 1,500 m; vi.1992; Paul Hanson; traps #1 + #2, Malaise. ***Paratypes*** Costa Rica • 2 ♀; same data as for holotype • 2 ♀; same collecting data as for preceding • 1 ♀; female; same collecting data as for preceding; 1,700 m; 11.iv.1993.

#### Diagnosis.

*Bohayella hansoni* sp. nov. can be distinguished from *B. geraldinae* sp. nov. by the following characters: apical maxillary palpomere slightly longer than fifth maxillary palpomere; median crenula of notauli ~ 0.38× longer than median crenula of scutellar sulcus; scutellar sulcus with three crenulae; hind basitarsus cylindrical; dorsal metasoma mostly melanistic.

#### Description.

**Figure 4.**
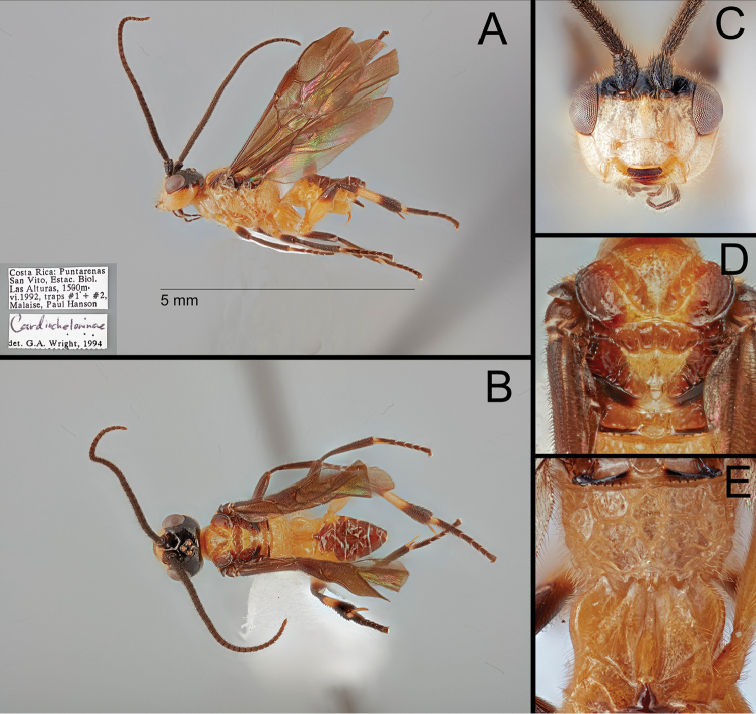
*B. hansoni* sp. nov., holotype. **A** lateral habitus **B** dorsal habitus **C** anterior head **D** dorsal mesonotum **E** dorsal propodeum and anterior metasoma.

**Female.** Body 3.9–4.1 mm. Forewing length: 3.9–4.1 mm Antenna length: 4.1–4.5 mm. ***Head*.** Antenna 32–34-segmented. Interantennal space with well-developed median carina. POL 1.22× longer than diameter of anterior ocellus (11:9) (Fig. 4B). Eye sparsely setose with short eye setae; length of eye ~ 0.86× longer than median width of gena in lateral view (31:36). Width of clypeus 2.00× longer than height (56:28) (Fig. 4C). Malar space 1.80× longer than basal width of mandible (36:20) (Fig. 4C). Mandible bidentate. Maxillary palpus 6-segmented; apical maxillary palpomere 1.31× longer than fifth maxillary palpomere (17:13). ***Mesosoma*.** Mesoscutum with sharp margin (Fig. 4B, D). Notauli broadly converging at base, with 11 crenulae; median crenula of notauli ~ 0.38× longer than median crenula of scutellar sulcus (6:16) (Fig. 4B, D). Scutellar sulcus with three crenulae (Fig. 4B, D). Postscutellar depression present (Fig. 4B, D). Propodeum rugulose, with well-defined median areola; median transverse carina on the propodeum reaching lateral margin (Fig. 4B, E). Pronotum dorso-posteriorly crenulate and antero-ventrally smooth. Mesopleuron dorsally and posteriorly with crenulate margin (Fig. 4A). Mesosternal sulcus broad and crenulate. Metapleuron rugulose. ***Legs*.** Basal spur on fore tibia ~ 0.87× longer than basitarsus (26:30). Width of hind femur ~ 0.30× longer than its length (33:111). Basal spur on hind tibia ~ 0.81× longer than basitarsus (58:72). Hind tarsal claw pectinate with four acute teeth. ***Wings*.** Forewing second submarginal cell trapezoidal, ~ 0.34× longer than its maximum width (26:77); 3r absent; Rs sharply angled at basal third; stigma ~ 2.82× longer than medial width (79:28). 1CUa short, 0.23× longer than 1Cub (11:47) (Fig. 4A). Hind wing 2-1A absent. ***Metasoma*.**T1 with a pair of lateral sutures posteriorly reduced, median length of T1 4.00× longer than apical width (56:14) (Fig. 4B, E). T2 with a ball-like projection, medially ~ 0.20× longer than T1 (11:56) (Fig. 4B, E). T3 ~ 2.55× longer than T2 medially (28:11) (Fig. 4B). Protruded ovipositor sheath ~ 0.20× longer than hind tibia and apically setose (26:129) (Fig. 4A).

**Color.** Body mostly pale; the following areas melanistic: antenna, vertex, frons, dorsal occiput, maxillary palpus, labial palpus, lateral mesonotal lobe (basally pale), lateral scutellum, margin of metanotum, apical fore femur, fore tibia, apical fore tarsus, apical mid femur, mid tibia, apical mid tarsus, apical hind femur, basal and apical hind tibia, apical hind tarsus, T2–T8, ovipositor sheath. Wings entirely infuscate, stigma entirely melanistic.

**Male.** Unknown.

#### Host.

Unknown.

#### Distribution.

**Figure 5.**
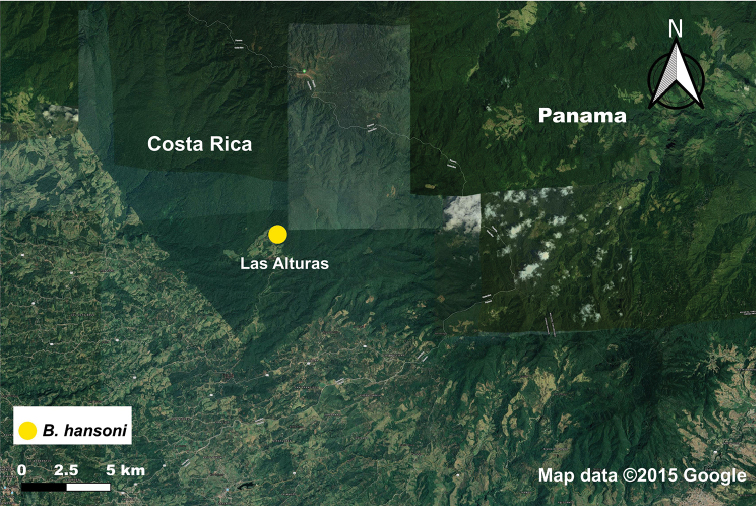
Distribution map of *B. hansoni* sp. nov. from Las Alturas Biological Research Station. Map data 2020 Google.

*Bohayella hansoni* sp. nov. is known only from the Las Alturas Biological research station owned and operated by Stanford University in Las Alturas, San Vito, Costa Rica at the elevations of 1,500 m and 1,700 m (Figs 1, 5).

#### Etymology.

This species is named in honor of Dr Paul Hanson, collaborator and professor at the Escuela de Biología, Universidad de Costa Rica. He worked tirelessly for many years collecting and sorting Costa Rican braconids from Malaise samples. SRS is very grateful for his dedication to Hymenoptera studies.

## Discussion

Most genus-level diagnostic characters are shared by both Old World and New World members (*B. geraldinae* sp. nov. and *B. hansoni* sp. nov.). None of the New World members have a mostly black body, 5-segmented maxillary palpi, or apically infuscate forewings. The following characters are only shared by New World members: angled Rs vein of forewing (Figs 3F, 4A), pectinate hind tarsal claw with sharp apical tooth (Fig. 3E), and antero-posteriorly slightly expanded hind basitarsus (Fig. 3A).

Specimens of *B. hansoni* sp. nov. collected at altitudes above 1,500 m have more melanistic metasoma than specimens of *B. geraldinae* sp. nov. collected at a low altitude of 100 m (Figs 3F, 4B). The melanism associated with high elevation was confirmed not only in braconid wasps such as members of the genus *Sendaphne* Nixon, 1965 (Nixon 1965) (Fernandez-Triana et al. 2014) and *Meteorus pulchricornis* (Wesmael, 1835) (Abe et al. 2013), but also in other hymenopteran insects such as members of a vespid species, *Agelaia pallipes* (Olivier, 1792) (de Souza et al. 2020) as well as an undescribed scelionid species of *Lapitha* Ashmead, 1893 (Mora and Hanson 2019). According to Abe et al. (2013), emerged adults of *M. pulchricornis* were more melanistic when cocoons were reared at lower temperatures, and the effects of the melanism resulted in increasing body temperatures and improved flight ability of adult *M. pulchricornis*. Melanism of *B. hansoni* sp. nov. at high elevations may induce similar outcomes as in *M. pulchricornis*. Further research is needed when enough live samples are available to confirm this.

The elevation of Costa Rica ranges from sea level to 3,819 m (Hanson and Gauld 1995). If additional sampling is conducted across the country and more species of *Bohayella* are discovered, altitudinal distribution patterns of members of Costa Rican *Bohayella* can be investigated in the future (e.g., Aguirre et al. 2018).

## Supplementary Material

XML Treatment for Bohayella

XML Treatment for Bohayella geraldinae

XML Treatment for Bohayella hansoni
